# Predicting and improving the protein sequence alignment quality by support vector regression

**DOI:** 10.1186/1471-2105-8-471

**Published:** 2007-12-03

**Authors:** Minho Lee, Chan-seok Jeong, Dongsup Kim

**Affiliations:** 1 Department of Bio and Brain Engineering, Korea Advanced Institute of Science and Technology, Daejeon, Republic of Korea

## Abstract

**Background:**

For successful protein structure prediction by comparative modeling, in addition to identifying a good template protein with known structure, obtaining an accurate sequence alignment between a query protein and a template protein is critical. It has been known that the alignment accuracy can vary significantly depending on our choice of various alignment parameters such as gap opening penalty and gap extension penalty. Because the accuracy of sequence alignment is typically measured by comparing it with its corresponding structure alignment, there is no good way of evaluating alignment accuracy without knowing the structure of a query protein, which is obviously not available at the time of structure prediction. Moreover, there is no universal alignment parameter option that would always yield the optimal alignment.

**Results:**

In this work, we develop a method to predict the quality of the alignment between a query and a template. We train the support vector regression (SVR) models to predict the MaxSub scores as a measure of alignment quality. The alignment between a query protein and a template of length *n *is transformed into a (*n *+ 1)-dimensional feature vector, then it is used as an input to predict the alignment quality by the trained SVR model. Performance of our work is evaluated by various measures including Pearson correlation coefficient between the observed and predicted MaxSub scores. Result shows high correlation coefficient of 0.945. For a pair of query and template, 48 alignments are generated by changing alignment options. Trained SVR models are then applied to predict the MaxSub scores of those and to select the best alignment option which is chosen specifically to the query-template pair. This adaptive selection procedure results in 7.4% improvement of MaxSub scores, compared to those when the single best parameter option is used for all query-template pairs.

**Conclusion:**

The present work demonstrates that the alignment quality can be predicted with reasonable accuracy. Our method is useful not only for selecting the optimal alignment parameters for a chosen template based on predicted alignment quality, but also for filtering out problematic templates that are not suitable for structure prediction due to poor alignment accuracy. This is implemented as a part in FORECAST, the server for fold-recognition and is freely available on the web at

## Background

As the number of protein sequences is exponentially growing, knowledge on their structures and functions is lagging far behind the growth rate of the number of new protein sequences because the experiments to determine structures and functions are difficult and time-consuming. One way to resolve this problem is computational methods such as structure and function prediction. In the case of protein structure prediction, computational methods fall into two categories; *ab initio *folding method and comparative modeling. *Ab initio *folding method is based on physical principles and does not require prior knowledge on protein structures, but comparative modeling [1] has shown superior performance throughout recent experiments assessing the effectiveness of structure prediction methods such as CASP (Critical Assessment of Structure Prediction) [2].

The first step in comparative modeling is the fold recognition in which one searches for homologous proteins with known structure and chooses the best one that can be used as a template. After this process, the alignment between the selected template and the query protein is generated. Finally the alignment is used to build the 3-dimensional structure models by using 3D model building tools such as MODELLER [1,3]. High-quality query-template alignments are, therefore, essential for successful homology modeling. Thus, there are two factors that essentially determine the quality of predicted protein structures; good templates and high quality query-template alignments. There have been many approaches to increase the performance of fold recognition. Progress in fold recognition has made it possible to increase the structural coverage of newly sequenced genomes [4] and to improve our ability to predict the protein structures as demonstrated in recent CASP experiments.

Importance of alignment accuracy for comparative modeling has been already addressed [5]. Among many sequence alignment methods, the easiest way is to use sequence-sequence alignments such as Smith-Waterman [6] or BLAST algorithm [7]. Other ways are to utilize evolutionary information: profile-sequence alignments such as PSI-BLAST [8] and sequence-profile alignments such as IMPALA [9]. To get better alignments, it has been shown in many studies that using profiles of both the query and the template, named profile-profile alignment, are superior to sequence-profile methods and profile-sequence methods [10]. Even though profile-profile alignments are better, they do not always provide the optimal alignments [11]. Profile-profile alignments can be carried out in many different ways [12-14] and the alignment results change as alignment options vary. There is no single best profile-profile method and the universal alignment option that always generates the optimal alignment.

To overcome this problem, some methods such as Consensus [15], ESyPred3D [16], Multiple Mapping Method (MMM) [17], and methods using genetic algorithm [18,19] have used population of suboptimal alignments. ESyPred3D fixes the redundant results from suboptimal alignments and finds optimal alignments by moving anchor point. Consensus make alignments by consensus of several alignments based on the consensus strength and by discarding the residues where alternative alignments differ. These two methods use limited number of alternative alignments. On the other hands, other two methods have used genetic algorithm to generate sub alignments as many as possible. After sets of model structure are constructed from alignments, score of each model is calculated by fitness function such as atom-atom potential [20] and Z-score [21]. However, these approaches take longer time, and alignments made by crossover are likely to be biologically meaningless. MMM, the recent study, focused on minimizing alignment errors based on its own scoring function by combining differently alignment segments from alternative alignments. MMM outperformed other methods and showed significant improvements.

We introduce here a novel method not only to predict the alignment quality but also to improve the alignment quality by support vector regression (SVR) [22]. Machine learning technique such as the artificial neural network (ANN) or support vector machine (SVM) [23] has been a popular tool for fold recognition, but is only available for feature vectors of fixed length. A new method in which all templates in template library have feature vectors of different lengths with profile-profile alignments scores has been recently developed [24]. In our work, a modified version has been used. Among many different kinds of measures for the alignment quality, MaxSub [25], which has been used as a measure in assessment experiments of structure prediction such as CASP [26], CAFASP [27], and LiveBench [28], is used to represent a measure of alignment quality. MaxSub is a good measure of alignment quality in that it is a normalized single numeric and reflects structure-level quality.

Our attempt to develop a method to predict the alignment quality is not entirely new. A related work [29] has been published, but the alignment quality prediction was not their final research goal. Rather, in the work by Xu [29], the predicted alignment quality was used to improve performance of fold recognition. In the present work, we develop a highly accurate method to predict the alignment quality, and we utilize the method not only to maximize the alignment quality and but also to choose good templates. In our work, an alignment of a query protein against its template of length *n *is converted into a feature vector of length *n *+ 1 composed of profile-profile alignment scores and the length of the query protein. The predicted MaxSub score is calculated by the SVR model specifically built for that template. The test results show highly accurate regression performance. For a pair of a query and a template, various alignments are generated by using many different combinations of alignment parameters. The SVR model for the template is then used to find the optimal alignment parameters which are specific to that pair. We name this method 'adaptive selection' method. The adaptive selection method outperforms the method which uses the universal alignment option for large-scale testing set.

## Results and discussion

### Performance measures of SVRs

**Figure 1:**
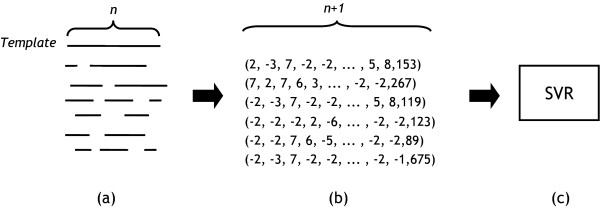
**Generation of the input feature vectors from alignments**. (a) The sequence of a template of length n is aligned to the sequences of examples by profile-profile alignment method. (b) Each alignment is transformed to (n + 1)-dimensional feature vector composed of the alignment scores at n positions and the total alignment score. (c) These feature vectors are used to train SVR model for the target template.

**Figure 2:**
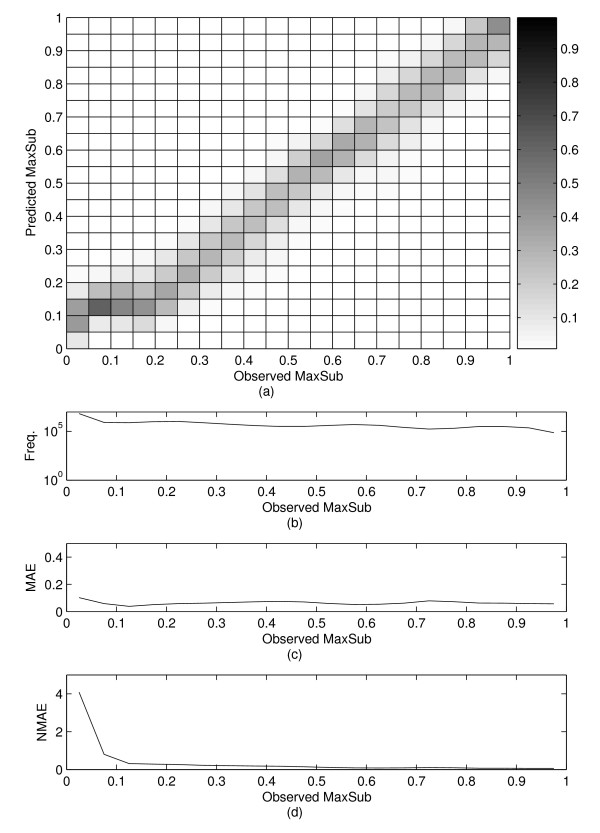
**Performance of SVR models**. (a) Correlations between observed and predicted MaxSub scores with correlation coefficient of 0.9453. Adjacent color bar shows the mapping of relative density. (b) Plot of frequency distribution. (c) Plot of MAE distribution. (d) Plot of NMAE distribution.

Alignments are converted into (*n *+ 1) dimensional feature vectors which are input of SVRs where *n *is the length of the templates (Figure 1). In order to evaluate the performance of the method, trained SVR models are evaluated for the testing set. The correlation between observed and predicted MaxSub values is presented in the density map (Figure 2a). Each column in the figure2a is normalized independently by dividing the number of alignments with a specific range of MaxSub scores by the total number of alignments in that column. The number of alignments in each column is plotted on Figure 2b. The highest density is represented by black squares; the lowest density is represented by white squares. The Pearson correlation coefficient is calculated from the pairs of predicted MaxSub scores and observed MaxSub scores. The calculated correlation coefficient is 0.945. A previous related work [29] has reported the correlation coefficient of 0.71, which is lower than that of the present method. However, because the testing set and the measure of alignment quality in the previous work (the measure of alignment quality was calculated by comparing the sequence alignment and the structural alignments generated by SARF [30] that were assumed to be the gold standard) are different from those used in this work, direct comparison between the two methods may not have much meaning, although much higher correlation coefficient of our work seems to suggest that the present method is apparently better at predicting the alignment quality than the previous method. The good correlation coefficient and the density diagram with good diagonal shape imply that the MaxSub scores as a measure of alignment quality can be accurately predicted. Moreover, the results suggest that for each query-template pair it is possible to find its own optimal alignment parameters that would maximize the alignment quality.

In addition to the Pearson correlation coefficient, three different measures of errors are also calculated. The first one is the mean absolute error (MAE) which is given by

MAE=1N∑i=1N|yi−oi|
 MathType@MTEF@5@5@+=feaafiart1ev1aaatCvAUfKttLearuWrP9MDH5MBPbIqV92AaeXatLxBI9gBaebbnrfifHhDYfgasaacPC6xNi=xI8qiVKYPFjYdHaVhbbf9v8qqaqFr0xc9vqFj0dXdbba91qpepeI8k8fiI+fsY=rqGqVepae9pg0db9vqaiVgFr0xfr=xfr=xc9adbaqaaeGacaGaaiaabeqaaeqabiWaaaGcbaGaeeyta0KaeeyqaeKaeeyrauKaeyypa0tcfa4aaSaaaeaacqaIXaqmaeaacqWGobGtaaGcdaaeWbqaamaaemaabaGaemyEaK3aaSbaaSqaaiabdMgaPbqabaGccqGHsislcqWGVbWBdaWgaaWcbaGaemyAaKgabeaaaOGaay5bSlaawIa7aaWcbaGaemyAaKMaeyypa0JaeGymaedabaGaemOta4eaniabggHiLdaaaa@43EA@

where *y*_*i *_is the predicted value, *o*_*i *_is the observed value, and N the total number of the predictions. The normalized MAE (NMAE) is defined as follows

NMAE=1N∑i=1N|yi−oi|oi.
 MathType@MTEF@5@5@+=feaafiart1ev1aaatCvAUfKttLearuWrP9MDH5MBPbIqV92AaeXatLxBI9gBaebbnrfifHhDYfgasaacPC6xNi=xI8qiVKYPFjYdHaVhbbf9v8qqaqFr0xc9vqFj0dXdbba91qpepeI8k8fiI+fsY=rqGqVepae9pg0db9vqaiVgFr0xfr=xfr=xc9adbaqaaeGacaGaaiaabeqaaeqabiWaaaGcbaGaeeOta4Kaeeyta0KaeeyqaeKaeeyrauKaeyypa0tcfa4aaSaaaeaacqaIXaqmaeaacqWGobGtaaGcdaaeWbqcfayaamaalaaabaWaaqWaaeaacqWG5bqEdaWgaaqaaiabdMgaPbqabaGaeyOeI0Iaem4Ba82aaSbaaeaacqWGPbqAaeqaaaGaay5bSlaawIa7aaqaaiabd+gaVnaaBaaabaGaemyAaKgabeaaaaaaleaacqWGPbqAcqGH9aqpcqaIXaqmaeaacqWGobGta0GaeyyeIuoakiabc6caUaaa@4952@

The last one is the root-mean-square error (RMSE) given by

RMSE=1N∑i=1N(yi−oi)2.
 MathType@MTEF@5@5@+=feaafiart1ev1aaatCvAUfKttLearuWrP9MDH5MBPbIqV92AaeXatLxBI9gBaebbnrfifHhDYfgasaacPC6xNi=xI8qiVKYPFjYdHaVhbbf9v8qqaqFr0xc9vqFj0dXdbba91qpepeI8k8fiI+fsY=rqGqVepae9pg0db9vqaiVgFr0xfr=xfr=xc9adbaqaaeGacaGaaiaabeqaaeqabiWaaaGcbaGaeeOuaiLaeeyta0Kaee4uamLaeeyrauKaeyypa0ZaaOaaaeaajuaGdaWcaaqaaiabigdaXaqaaiabd6eaobaakmaaqahabaWaaeWaaeaacqWG5bqEdaWgaaWcbaGaemyAaKgabeaakiabgkHiTiabd+gaVnaaBaaaleaacqWGPbqAaeqaaaGccaGLOaGaayzkaaWaaWbaaSqabeaacqaIYaGmaaaabaGaemyAaKMaeyypa0JaeGymaedabaGaemOta4eaniabggHiLdaaleqaaOGaeiOla4caaa@45BD@

MAE, NMAE, and RMSE are 0.0775, 1.877, and 0.0969, respectively, also shown in Table 1 and distributions of MAE and NMAE are shown in Figure 2c and Figure 2d, respectively. MAE is always lower than 0.2 for all the range of observed MaxSub scores when the window size is set to 0.5.

**Table 1:** Performance of SVR models for overall test set and at three levels of SCOP hierarchy. Pearson stands for Pearson correlation coefficient. MAE, NMAE, and RMSE are types of error

	All	Family	Superfamily	Fold
Pearson	0.9453	0.9185	0.8318	0.6106
MAE	0.0775	0.0630	0.0773	0.0848
NMAE	1.8771	0.3112	1.8344	2.6738
RMSE	0.0969	0.0936	0.0962	0.0988

### Adaptive selection of the alignment options having the best MaxSub score

The ultimate objective of predicting alignment quality is to find the best alignment. One straightforward, although not the best, way to do this is to choose a set of the optimal alignment parameters, such as gap opening penalty, gap extension penalty, baseline score, and the amount of secondary structure term, that would yield the best alignments overall. However, as seen in Table 2 where the average MaxSub scores for the alignments generated with various different combination of the alignment parameters are shown, there is no such single set of parameters that are universally optimal for all query-template pairs. For example, for the query-template protein pairs that are related at the family level, the optimal alignment parameters are 9, 1, 1, and 0.5 for gap opening penalty, gap extension penalty, baseline score, and the secondary structure information, respectively, while those parameters change to 12, 2, 0, and 2 for the protein pairs that are related at the fold level. Overall, the maximum of average MaxSub scores is 0.2386 with the optimal alignment parameters of 9, 1, 1, and 1, which interestingly are not the optimal parameters for the protein pairs related at any level of similarity.

**Table 2:** Average MaxSub scores of the alignments generated by using various combinations of alignment parameters for the protein pairs related at the three SCOP levels. Open, Extension, and Baseline column shows gap open penalty, gap extension penalty and baseline value, respectively. '2nd' stands for the weight of predicted secondary structure. The best option showing highest MaxSub at each level is bolded.

				Average MaxSub			
				
Open	Extension	Baseline	2nd	All	Family	Superfamily	Fold
5	1	0	0	0.2104	0.5930	0.1636	0.0447
5	1	1	0	0.2172	0.6073	0.1679	0.0492
5	2	0	0	0.2130	0.6062	0.1566	0.0477
5	2	1	0	0.2105	0.6060	0.1494	0.0470
9	1	0	0	0.2200	0.6080	0.1774	0.0488
9	1	1	0	0.2208	0.6133	0.1716	0.0514
9	2	0	0	0.2171	0.6115	0.1621	0.0505
9	2	1	0	0.2131	0.6104	0.1508	0.0494
13	1	0	0	0.2176	0.6096	0.1696	0.0479
13	1	1	0	0.2158	0.6109	0.1609	0.0487
13	2	0	0	0.2131	0.6102	0.1530	0.0481
13	2	1	0	0.2088	0.6076	0.1429	0.0467
5	1	0	1	0.2210	0.5950	0.1705	0.0619
5	1	1	1	0.2298	0.6070	0.1784	0.0696
5	2	0	1	0.2283	0.6066	0.1738	0.0696
5	2	1	1	0.2286	0.6089	0.1713	0.0706
9	1	0	1	0.2342	0.6129	0.1853	0.0718
9	1	1	1	**0.2386**	0.6176	0.1877	0.0771
9	2	0	1	0.2373	0.6175	0.1837	0.0770
9	2	1	1	0.2345	0.6169	0.1770	0.0755
13	1	0	1	0.2355	0.6139	0.1851	0.0741
13	1	1	1	0.2374	0.6165	0.1852	0.0767
13	2	0	1	0.2356	0.6163	0.1808	0.0759
13	2	1	1	0.2319	0.6143	0.1730	0.0737
5	1	0	2	0.2111	0.5765	0.1572	0.0586
5	1	1	2	0.2208	0.5935	0.1629	0.0669
5	2	0	2	0.2216	0.5950	0.1609	0.0691
5	2	1	2	0.2248	0.6017	0.1611	0.0725
9	1	0	2	0.2247	0.6014	0.1676	0.0684
9	1	1	2	0.2311	0.6090	0.1719	0.0754
9	2	0	2	0.2324	0.6101	0.1717	0.0777
9	2	1	2	0.2327	0.6106	0.1706	0.0788
13	1	0	2	0.2290	0.6073	0.1713	0.0723
13	1	1	2	0.2337	0.6110	0.1750	0.0780
13	2	0	2	0.2343	0.6122	0.1741	**0.0793**
13	2	1	2	0.2332	0.6120	0.1707	0.0792
5	1	0	0.5	0.2214	0.5979	0.1738	0.0593
5	1	1	0.5	0.2288	0.6094	0.1801	0.0652
5	2	0	0.5	0.2260	0.6095	0.1729	0.0638
5	2	1	0.5	0.2247	0.6104	0.1680	0.0635
9	1	0	0.5	0.2337	0.6141	**0.1888**	0.0678
9	1	1	0.5	0.2359	**0.6183**	0.1881	0.0709
9	2	0	0.5	0.2328	0.6174	0.1807	0.0693
9	2	1	0.5	0.2291	0.6170	0.1717	0.0672
13	1	0	0.5	0.2329	0.6150	0.1856	0.0678
13	1	1	0.5	0.2323	0.6162	0.1809	0.0687
13	2	0	0.5	0.2295	0.6160	0.1739	0.0672
13	2	1	0.5	0.2251	0.6139	0.1645	0.0647
Mean				0.2261	0.6090	0.1713	0.0651

The results suggest the following alignment strategy. Instead of using single universal set of alignment parameters for all query-template pairs, by simply picking up a different set of the alignment parameters that are uniquely optimal for a query-template pair, the alignment can be improved. If we do so, as seen in Table 3, the average of the overall MaxSub scores improves from 0.2386 to 0.2887 (0.0501 point improvement, corresponding to roughly 21% improvement).

**Table 3:** Comparison of average MaxSub scores. The values in the first row "Overall best option" are retrieved from Table 2.

	Average MaxSub			
	
Method	All	Family	Superfamily	Fold
Overall Best Option	0.2386	0.6176	0.1877	0.0771
Always Best (Upper Limit)	0.2887	0.6414	0.2505	0.1396
Adaptive Selection (Observed)	0.2563	0.6255	0.2128	0.0953
Adaptive Selection (Predicted)	0.3039	0.6385	0.2501	0.1669

Obviously, we do not know a priori which set of alignment parameters is optimal for a given query-template pair because the structure of a query protein is not known. Therefore, here we propose the 'adaptive selection' method. The adaptive selection procedure is carried out as follows. (1) Generate the alignments using many different combinations of alignment parameters. (2) Predict MaxSub scores of alignments using the trained SVR models. (3) Select the alignment that gives the highest predicted MaxSub score.

When we follow the adaptive selection procedure, the average of actual MaxSub scores of the alignments selected by the adaptive selection procedure improves to 0.2563 (Table 3), which corresponds to 0.0177 point or 7.42% improvement, compared to the single best option procedure. This improvement is statistically significant (p-value < 10^-300 ^calculated by Wilcoxon signed rank test [31]). It also indicates that the adaptive selection method can scoop roughly 35.3% (0.0177 vs. 0.0501) of the maximum improvement that can be achievable by selecting the optimal alignment parameters unique to each query-template pair. Moreover, it also implies that it is possible to improve the alignment quality even more by developing more accurate alignment quality prediction method.

### Performance at three levels of SCOP hierarchy

In this section, we describe performance at three levels of SCOP hierarchy (family, superfamily, and fold) to closely examine where the improvement is achieved. All the experiments carried out in the previous section are done for testing sets at the three different levels.

The density diagram in Additional file 1 shows the correlation at the family level. It looks similar to Figure 2a except that it shows weak correlation in low MaxSub score region. The reason seems to be that alignments of pairs at the family level likely have high MaxSub scores, and SVR models have not experienced sufficient alignments that have low MaxSub scores during the training stage. The correlation coefficient, MAE, NMAE and RMSE is 0.9185, 0.0630, 0.3112 and 0.0936, respectively (Table 1). Additional file 1 shows the number of alignments in different regions of observed MaxSub score. Additional file 2 shows the correlation at the superfamily level. It shows rather weak correlation in high MaxSub score region. The correlation coefficient, MAE, NMAE and RMSE is 0.8318, 0.0773, 1.8344 and 0.0962, respectively (Table 1). Contrary to the case of the family level, there are not many examples in high observed MaxSub region, which is the reason for weak correlation in high score region. The density map in Additional file 3 represents the correlation at the fold level. The correlation coefficient, MAE, NMAE and RMSE is 0.6106, 0.0848, 2.6738 and 0.0988, respectively (Table 1). Like the case of the superfamily level, it seems to show weak correlation at high score region.

In Table 2, the MaxSub scores are presented at three different levels. The averages are 0.6090, 0.1713, and 0.0651, and the values for best options are 0.6183, 0.1888, and 0.0793 at the level of family, superfamily, and fold, respectively. These values are also compared with corresponding scores achieved by adaptive selection method (Table 3). It is also observed that adaptive selection method shows higher performance at the every SCOP level as for overall testing set showing an improvement of 1.16, 12.7, and 20.2% at the family, superfamily and fold level, respectively.

**Figure 3:**
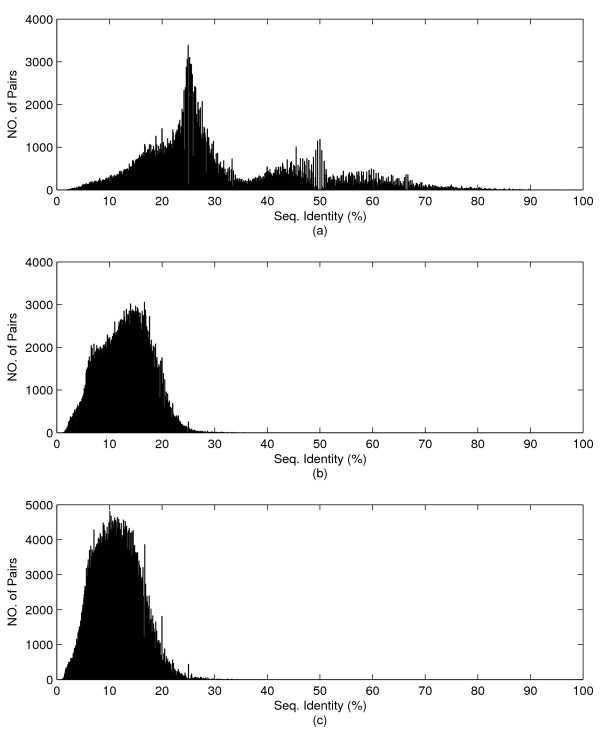
**Distribution of sequence identities on the test set**. Distribution of sequence identities of the query-template pairs on the test set at (a) family (b) superfamily (c) fold level.

To check diversity of test set, sequence identities of query-template pairs are presented in Fig at each SCOP level, family (Figure 3a), superfamily (Figure 3b), and fold (Figure 3c). The average values of sequence identity are 30.95%, 13.03%, and 11.51% at each SCOP level, respectively. Except for some pairs in the test set at family level, the sequence identities of almost all pairs are under 35%, "twilight zone [32]." The distribution tells our results are not based on high sequence identity.

### Alignments of pairs that are not related

**Figure 4:**
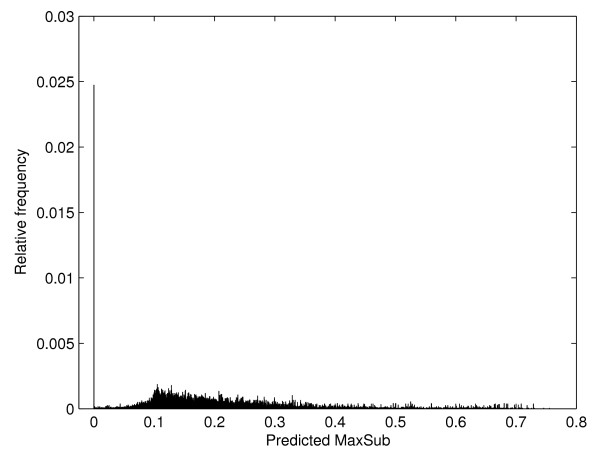
Histogram of predicted MaxSub scores of the alignments of the pairs that are not related at the fold level.

All protein pairs in the testing set used in the experiments share the similar structure at least at the fold level (see Methods). It is, therefore, necessary to check whether the trained SVR models show reliable performance for proteins which do not share the same fold. In order to check this, 10 unrelated proteins per each template are randomly selected, aligned against the templates, and transformed into feature vectors. The vectors are then applied to SVR models of the templates to predict MaxSub scores. All the observed MaxSub scores are zero without exception. Thus all the predicted values should be zero in ideal situation. Histogram of predicted values is shown in Figure 4. Unfortunately, most predicted values are not zero. The mean is 0.1979 and the standard deviation is 0.1257. We can infer here that SVR models predict the MaxSub scores larger than the true values in low MaxSub score region. The histogram shows that the true MaxSub scores of alignments predicted to have MaxSub score near 0.1 might be zero. Thus, if a predicted MaxSub is low and is not zero, it should be carefully examined.

### Alignments of the pairs whose MaxSub scores are zero despite being in the same family

**Figure 5:**
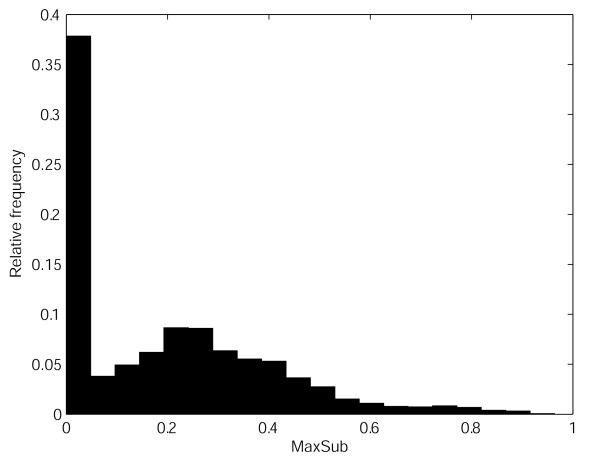
Histogram of MaxSub scores by adaptive selection method for the alignments of the pairs sharing the same family whose MaxSub score is zero when single best alignment option method is used.

It is expected that two proteins in the same SCOP family have a similar 3D structure. There are, however, many alignments of the pairs in the same family for which observed MaxSub scores are zero (Additional file 1). When MaxSub score is zero, the alignment is completely incorrect by definition [25]. For these pairs, we check how much improvement can be achieved by adaptive selection method. Figure 5 shows histogram of MaxSub scores which is given by adaptive selection method for the alignments of those pairs. For about 37.3% of all pairs, there is no improvement, while about 62.7% of pairs achieve some improvement. In other words, around 63% of completely incorrect alignments between a pair of protein related at the family level are corrected into partially corrected alignments by changing alignment options by adaptive selection method.

Then, what are the reasons that remaining 37.3% of pairs gain no improvement? The most obvious one is regression error. Adaptive selection method might wrongly select an option due to regression error although there is another option that might give improved MaxSub score. When we examine the data, it appears that 17.9% constitute this type. Second, it may result from the limitation of profile-profile alignments. It has been well known that profile-profile alignment is not always the optimal alignment when compared to the structure alignment. It may fail to align a query against a particular template with any alignment options due to problem of alignment method itself. The third reason may be the lack of alignment options in our method. Although 48 options are used in our work, they may not be sufficient because the options used here do not cover all possible cases. For example, to align a particular pair of proteins, abnormally large gap open penalty might be necessary.

The fourth reason may be the limitation of MaxSub score as a measure of alignment quality. There have been a number of assessment methods for alignment quality. It has been controversial what evaluation method is the best. There are many alternative measures such as GDT_TS [33,34], LGscore [35] and MAMMOTH [36]. Another aspect is that MaxSub score is basically sequence-dependent assessment. In sequence-dependent assessment, only corresponding residues in alignment are compared. It is stricter than sequence-independent assessment [37,38] for alignments which are slightly shifted from the optimal alignment, which might make MaxSub scores of some alignments become zero. Our method might be improved by combining these sequence-dependent and sequence-independent methods.

**Figure 6:**
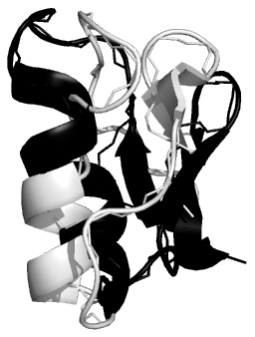
**Superposition of 2 SCOP domains**. Superposition of SCOP domain d1tsk__ (bright) onto d1chl__ (dark), both of which belong to the same family (g.3.7.2).

Finally, some template structures may not be good for predicting the structure of a query protein, even though they are in the same family with a query protein. One example of this case is an alignment of a query protein, d1tsk__, against a template, d1chl__, both of which belong to the same family (g.3.7.2). All MaxSub scores of the alignments generated by using all 48 options are zero. To check whether it is caused by the problem of profile-profile method, we perform the structural alignment by CE algorithm [39], and we find that the MaxSub score of this structural alignment is also zero. Figure 6 shows a superposition of these two proteins. It can be inferred that there are bad templates for structure prediction although they are the same family member with a query protein. It might be caused by strict definition of MaxSub. However, in the view of MaxSub, the template d1chl__ is apparently a bad one for the query.

**Figure 7:**
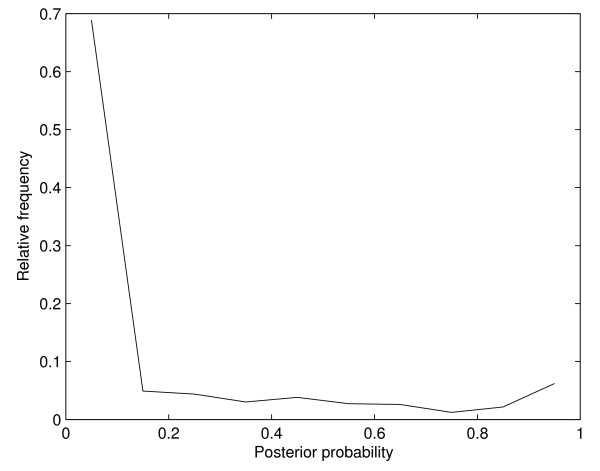
Distribution of posterior probabilities of outputs of SVM for fold-recognition.

Such alignments are tested by the fold recognition method developed in the previous study [24] to see their fold recognition scores. The raw SVM outputs are converted into posterior probabilities [40], ranging from zero to one, and the distribution of these probabilities is shown in Figure 7. The distribution exhibits two peaks, near zero and one. If we choose decision-threshold as 0.5, roughly 15% of pairs are classified into protein pairs sharing the same family. Let us consider a situation where one tries to predict the protein structure and chooses the templates by means of fold-recognition score only. For some cases, if a certain template is selected simply because it is predicted to be homologous at the family level, the final result of structure prediction might be failed due to wrong selection of the template. Adaptive selection method may help to filter this sort of templates out and can prevent ones from selecting these bad templates.

### Benchmark test

The benchmark test of adaptive selection method is carried out on 62 targets of CASP7. We use EsyPred3D and Multiple Mapping Method (MMM) for the comparing. Both are publicly available web servers, and alignments and 3D models are provided. We used the default options of the servers.

Out of all 88 targets of CASP7, 77 targets have significantly close template in SCOP 1.69 according to the result of fold search by Proteinsilico [41]. The templates of 62 targets of those are trained in our dataset, and these target-template pairs are used in the benchmarking.

Table 4 shows the performances of MMM, EsyPred3D, and adaptive selection method. The greatest values of MaxSub, Mammoth Z-score, TM-score [42], GDT_TS for each pair are bolded. Our method gives better alignments having larger MaxSub than other two methods on average (0.264 vs. 0.203 and 0.182). In the aspect of other measures the adaptive method also shows the best performance. In addition, the values of our method are statistically significant according to p-values calculated by Wilcoxon signed rank test [31] with significance level 0.05.

**Table 4:** Alignment performances on CASP 7 using MMM, ESyPred3D, and Adaptive method. The highest value for each pair is bolded. P-values are calculated by Wilcoxon signed rank test.

		MaxSub			Mammoth Z-score			TM-score			GDT_TS		
		
Target	Template	MMM	ESyPred3D	Adaptive Method	MMM	ESyPred3D	Adaptive Method	MMM	ESyPred3D	Adaptive Method	MMM	ESyPred3D	Adaptive Method
T0283	1pq1a_	0.000	0.000	**0.212**	2.30	1.18	**4.69**	0.234	0.216	**0.315**	0.210	0.208	**0.301**
T0288	1r6ja_	0.743	0.668	**0.756**	12.42	**12.81**	12.56	**0.775**	0.720	0.767	0.769	0.712	**0.769**
T0289	1boub_	0.000	0.000	0.000	**1.36**	1.13	0.48	**0.186**	0.160	0.179	0.073	0.070	**0.075**
T0291	1rdqe_	**0.515**	0.483	0.484	**28.76**	28.03	27.82	**0.735**	0.691	0.700	**0.568**	0.542	0.538
T0292	1rdqe_	0.565	**0.579**	0.556	27.90	28.48	**29.66**	0.768	**0.786**	0.774	0.617	**0.639**	0.631
T0293	1jg1a_	0.221	0.000	**0.240**	**12.65**	11.89	12.20	**0.405**	0.175	0.385	0.290	0.082	**0.300**
T0295	1jg1a_	0.000	0.212	**0.288**	0.23	8.82	**11.94**	0.171	0.252	**0.367**	0.093	0.208	**0.299**
T0296	1p1xa_	0.048	0.000	**0.055**	2.40	3.68	**5.78**	**0.216**	0.175	0.194	0.077	0.068	**0.091**
T0297	1k7ca_	0.199	0.216	**0.466**	17.17	**18.46**	16.71	0.421	0.425	**0.596**	0.289	0.299	**0.493**
T0299	1p5fa_	0.000	0.000	0.000	0.09	0.36	**0.40**	**0.192**	0.159	0.170	**0.132**	0.107	0.113
T0300	1rh5b_	**0.256**	0.000	0.234	3.83	2.19	**4.32**	**0.276**	0.239	0.249	**0.346**	0.258	0.289
T0302	1orja_	0.000	0.000	**0.149**	1.72	**1.92**	1.72	0.257	0.224	**0.265**	0.210	0.189	**0.239**
T0303	1o08a_	**0.520**	0.517	0.425	**25.55**	25.54	25.48	**0.743**	0.718	0.666	**0.607**	0.588	0.554
T0304	1j3wa_	**0.192**	0.000	0.000	0.51	1.91	**2.79**	**0.301**	0.140	0.200	**0.280**	0.126	0.220
T0305	1lyva_	0.440	0.410	**0.451**	26.72	22.98	**26.97**	**0.705**	0.568	0.668	**0.532**	0.444	0.522
T0308	1f4pa_	**0.181**	0.000	0.156	8.29	1.92	**8.78**	**0.396**	0.139	0.348	**0.289**	0.094	0.247
T0310	1us6a_	0.000	0.000	0.000	1.03	0.79	**2.83**	**0.085**	0.055	0.060	**0.049**	0.041	0.032
T0315	1i0da_	0.388	0.296	**0.457**	25.66	18.39	**26.55**	0.667	0.582	**0.720**	0.476	0.394	**0.541**
T0316	1kqpa_	0.140	0.115	**0.169**	10.72	**15.53**	13.13	**0.316**	0.227	0.277	0.170	0.146	**0.190**
T0317	1byi__	**0.174**	0.000	0.169	0.85	**8.44**	6.16	**0.314**	0.246	0.269	**0.237**	0.169	0.212
T0318	1rtqa_	0.048	0.098	**0.097**	6.47	5.48	**16.72**	0.191	**0.256**	0.251	0.070	0.117	**0.130**
T0321	1jbea_	0.000	0.000	0.000	**2.77**	1.82	1.81	**0.166**	0.155	0.146	0.090	0.081	**0.090**
T0322	1vh5a_	**0.614**	0.596	0.603	16.48	16.95	**17.75**	**0.707**	0.673	0.698	0.622	0.599	**0.629**
T0323	1c20a_	0.000	0.000	0.000	1.01	0.58	**1.62**	**0.173**	0.118	0.114	**0.103**	0.080	0.083
T0324	1o08a_	0.562	0.562	**0.582**	**25.93**	24.76	24.84	0.748	**0.760**	0.750	0.604	0.626	**0.629**
T0325	1i0da_	0.101	0.000	**0.103**	**13.96**	4.04	5.39	0.322	0.221	**0.395**	0.183	0.120	**0.217**
T0326	1p5fa_	0.065	0.131	**0.190**	8.81	7.48	**14.35**	0.220	0.241	**0.349**	0.112	0.148	**0.238**
T0328	1mwqa_	0.000	0.000	**0.089**	0.42	3.73	**9.59**	0.126	0.095	**0.163**	0.060	0.049	**0.110**
T0329	1o08a_	0.464	**0.471**	**0.471**	**25.57**	25.29	24.67	**0.683**	0.655	0.661	**0.545**	0.529	0.529
T0330	1o08a_	**0.424**	0.365	0.376	**27.13**	21.07	22.94	**0.694**	0.649	0.604	**0.552**	0.519	0.496
T0332	1io0a_	0.000	0.000	**0.117**	3.31	1.66	**3.78**	0.235	0.180	**0.254**	0.178	0.137	**0.193**
T0335	1hz4a_	0.000	0.000	**0.458**	1.76	3.50	**3.80**	0.267	0.312	**0.377**	0.464	0.476	**0.542**
T0338	1tqga_	0.000	0.000	0.000	0.71	1.92	**2.02**	**0.148**	0.121	0.101	**0.093**	0.089	0.090
T0339	1lc5a_	0.177	0.119	**0.237**	20.18	20.52	**26.16**	0.476	0.417	**0.531**	0.247	0.207	**0.316**
T0340	1r6ja_	0.697	0.740	**0.746**	**13.45**	11.95	12.35	0.743	0.755	**0.762**	0.742	0.742	**0.758**
T0341	1qcza_	**0.088**	0.000	0.079	**1.72**	1.35	1.38	**0.203**	0.141	0.163	**0.110**	0.067	0.107
T0353	2igd__	0.255	0.260	**0.280**	5.93	5.68	**6.84**	0.315	0.318	**0.339**	0.338	0.347	**0.365**
T0354	1fm0e_	0.000	0.000	0.000	1.11	**4.69**	1.25	**0.230**	0.191	0.220	**0.211**	0.164	0.209
T0356	1j27a_	0.000	0.000	0.000	-0.24	-0.87	**0.46**	0.083	**0.087**	0.045	0.034	**0.040**	0.031
T0357	1nxja_	0.000	0.000	**0.220**	**10.80**	5.39	6.19	0.221	0.186	**0.294**	0.169	0.129	**0.278**
T0359	1r6ja_	0.677	0.629	**0.683**	**13.48**	11.68	12.35	**0.718**	0.663	0.701	**0.707**	0.634	0.699
T0361	1t7ra_	0.126	0.112	**0.144**	**4.05**	1.21	1.98	0.221	**0.225**	0.192	**0.185**	0.173	0.173
T0362	1vh5a_	0.000	0.000	**0.449**	10.45	2.05	**13.78**	0.167	0.187	**0.538**	0.115	0.162	**0.477**
T0363	2igd__	0.000	0.000	**0.321**	**4.66**	4.02	4.02	0.176	0.156	**0.343**	0.178	0.166	**0.372**
T0364	1vh5a_	0.220	0.253	**0.489**	12.88	7.84	**14.39**	0.346	0.312	**0.557**	0.276	0.271	**0.508**
T0365	1g73a_	**0.146**	0.000	0.109	**5.23**	4.47	4.83	**0.247**	0.162	0.190	**0.188**	0.115	0.140
T0366	1r6ja_	0.685	0.754	**0.781**	**12.59**	11.78	11.37	0.722	0.774	**0.777**	0.702	**0.780**	0.765
T0367	1ug7a_	0.000	0.000	**0.220**	**7.65**	4.20	7.16	0.274	0.196	**0.304**	0.242	0.172	**0.264**
T0368	1hz4a_	**0.211**	0.159	0.208	**5.65**	4.49	4.16	**0.327**	0.295	0.308	0.261	0.239	**0.266**
T0369	1orja_	**0.166**	0.000	0.000	4.12	2.40	**5.12**	**0.234**	0.148	0.167	**0.225**	0.126	0.157
T0371	1f4pa_	0.000	0.000	0.000	0.34	2.51	**5.31**	**0.158**	0.154	0.144	0.078	**0.089**	0.086
T0372	1m44a_	0.000	0.000	**0.133**	0.04	0.14	**0.55**	0.143	0.131	**0.246**	0.064	0.057	**0.168**
T0373	1rh5b_	0.000	0.000	0.000	-0.06	**3.79**	3.02	0.132	0.147	**0.153**	0.125	**0.155**	0.154
T0374	1m44a_	0.293	**0.386**	0.384	13.33	**15.66**	14.94	0.445	**0.566**	0.545	0.361	**0.463**	0.459
T0375	1bx4a_	0.482	0.457	**0.508**	**32.34**	29.72	29.86	**0.741**	0.696	0.730	**0.542**	0.510	0.541
T0376	1twda_	0.125	0.108	**0.191**	14.05	**15.93**	15.39	0.303	0.346	**0.382**	0.167	0.189	**0.261**
T0378	1sdsa_	0.089	0.000	**0.159**	2.02	0.35	**8.18**	0.148	0.122	**0.227**	0.095	0.071	**0.177**
T0379	1o08a_	0.299	0.360	**0.403**	15.24	**15.96**	15.46	0.456	0.536	**0.569**	0.353	0.415	**0.455**
T0381	1tf1a_	0.534	**0.586**	0.582	21.04	**23.79**	23.68	0.629	**0.655**	0.651	0.537	**0.565**	0.560
T0382	1kpsb_	0.000	**0.171**	0.000	1.65	1.30	**3.08**	0.263	**0.280**	0.240	0.229	**0.254**	0.238
T0384	1jg1a_	0.000	0.000	0.000	0.16	1.08	**1.67**	**0.180**	0.140	0.127	0.068	**0.084**	0.083
T0385	1lb3a_	0.462	0.489	**0.659**	14.60	17.50	**18.96**	0.633	0.618	**0.759**	0.546	0.570	**0.659**
mean		0.203	0.182	0.264	9.564	9.086	10.712	0.367	0.338	0.391	0.292	0.273	0.328
p-value		1.042E-04	2.163E-07	-	3.8E-03	5.761E-05	-	0.2133	1.898E-06	-	9.996E-04	2.209E-08	-

## Conclusion

In the process of protein sequence alignment, generally only one particular set of alignment parameters is used throughout the all protein pairs, regardless of their evolutionary relationship. In some cases, many alignments are generated using many different combinations of alignment parameters, and then the potentially optimal alignment is chosen purely based on experience or intuition. In this work, however, we select the alignment parameters which are predicted to give the highest MaxSub score specific to a pair of a query and a template. Our work is distinguishable to other efforts to improve the quality of protein sequence alignments in that we directly predict alignment quality with quite good accuracy. By predicting the alignment quality and then choosing the optimal alignment parameters based on the prediction, we show that the alignment quality can be improved significantly. Our method can be utilized to select not only the optimal alignment parameters for a chosen template but also good templates with which the structure of a query protein can be best predicted.

In summary, we develop a method to predict the MaxSub score as an alignment quality of a given profile-profile alignment between a query and a template. The alignment between a query protein and a template of length n is transformed into a (*n *+ 1)-dimensional feature vector. These feature vectors are used to train the SVR models for the templates. We rigorously test the performance of the method using various evaluation measures such as Pearson correlation coefficient, MAE, NMAE, and RMSE. Results show the high correlation coefficient of 0.945 and low prediction errors. Trained SVR models are then applied to select the best alignment option which is chosen specifically to the pair of a query and a template. This adaptive selection procedure results in 7.4% improvement of MaxSub scores, compared to the scores when single best option is used for the all query-template pairs.

## Methods

### Data

To make a template library, classification by the SCOP version 1.69 [43] is used. First, the fold library composed of ~11,130 domains is constructed using domain subsets with less than 90% sequence identity to each other prepared by ASTRAL Compendium [44]. We choose the folds containing at least 20 members for training and testing the SVR models. A total of 7509 domains in 122 folds are selected as a result. Two thirds are used to train and the rest is used to test. To estimate the performance, we employ the three-fold cross-validation procedure.

### MaxSub score as alignment quality (target of each SVR)

Conventionally, the alignment quality is calculated by comparing the sequence alignment and the structural alignments generated by various structure alignment programs such as SARF [30], CE and MAMOTH, assuming that the structure alignments are the gold standard. A problem of this approach is that depending on the specific choice of structure alignment program, the structure alignments can vary significantly, especially for distant homolog pairs. A different approach is that first the structure prediction model of a query protein is quickly generated by directly copying C-α positions of all aligned residues of the template protein using the sequence alignment, and then the protein structure model quality measure such as MaxSub [25] or TM-score [42] is calculated and used as a alignment quality score. The second approach is more relevant to the present study, because the main focus of this work is how to generate good sequence alignments that would eventually lead to better structure models. Specifically, we use MaxSub [25], a popular model quality measure which finds the largest subset of C_α _atoms of a model that superimpose well over the experimental structure. At the stage of training, each alignment is converted into a structure model of the query protein. MaxSub score is then calculated using the model derived from the alignment and the correct structure, with *d *parameter set to 3.5 Å which has been found to be a good choice for the evaluation of fold-recognition models [25]. We have also considered to use TM-score [42], another popular model quality measure, as the alignment quality measure. However, it turned out that the correlation between MaxSub scores and TM-scores was as high as 0.95. Therefore, we expect that our specific choice of MaxSub score as the alignment quality measure does not affect the performance of our method and the main conclusion of this work.

### Profile-profile alignments and SVR feature vectors

To train SVR models for all templates in the training set, feature vector scheme developed in previous work [24] is adopted with slight modification. We first generate all-against-all alignments within the set sharing the same fold by profile-profile alignment scheme with 48 different combinations of alignment parameters (gap open-penalty, gap extension-penalty, base-line score, and weight of predicted secondary structure). The profile-profile alignment score to align the position *i *of a query *q *and the position *j *of a template *t *is given by

mij=∑k=120[fikqSjkt+Sikqfjkt]+sij+b
 MathType@MTEF@5@5@+=feaafiart1ev1aaatCvAUfKttLearuWrP9MDH5MBPbIqV92AaeXatLxBI9gBaebbnrfifHhDYfgasaacPC6xNi=xI8qiVKYPFjYdHaVhbbf9v8qqaqFr0xc9vqFj0dXdbba91qpepeI8k8fiI+fsY=rqGqVepae9pg0db9vqaiVgFr0xfr=xfr=xc9adbaqaaeGacaGaaiaabeqaaeqabiWaaaGcbaGaemyBa02aaSbaaSqaaiabdMgaPjabdQgaQbqabaGccqGH9aqpdaaeWbqaamaadmaabaGaemOzay2aa0baaSqaaiabdMgaPjabdUgaRbqaaiabdghaXbaakiabdofatnaaDaaaleaacqWGQbGAcqWGRbWAaeaacqWG0baDaaGccqGHRaWkcqWGtbWudaqhaaWcbaGaemyAaKMaem4AaSgabaGaemyCaehaaOGaemOzay2aa0baaSqaaiabdQgaQjabdUgaRbqaaiabdsha0baaaOGaay5waiaaw2faaaWcbaGaem4AaSMaeyypa0JaeGymaedabaGaeGOmaiJaeGimaadaniabggHiLdGccqGHRaWkcqqGZbWCdaWgaaWcbaGaeeyAaKMaeeOAaOgabeaakiabgUcaRiabdkgaIbaa@59BD@

where fikq
 MathType@MTEF@5@5@+=feaafiart1ev1aaatCvAUfKttLearuWrP9MDH5MBPbIqV92AaeXatLxBI9gBaebbnrfifHhDYfgasaacPC6xNi=xH8viVGI8Gi=hEeeu0xXdbba9frFj0xb9qqpG0dXdb9aspeI8k8fiI+fsY=rqGqVepae9pg0db9vqaiVgFr0xfr=xfr=xc9adbaqaaeGacaGaaiaabeqaaeqabiWaaaGcbaGaemOzay2aa0baaSqaaiabdMgaPjabdUgaRbqaaiabdghaXbaaaaa@317A@, fjkt
 MathType@MTEF@5@5@+=feaafiart1ev1aaatCvAUfKttLearuWrP9MDH5MBPbIqV92AaeXatLxBI9gBaebbnrfifHhDYfgasaacPC6xNi=xH8viVGI8Gi=hEeeu0xXdbba9frFj0xb9qqpG0dXdb9aspeI8k8fiI+fsY=rqGqVepae9pg0db9vqaiVgFr0xfr=xfr=xc9adbaqaaeGacaGaaiaabeqaaeqabiWaaaGcbaGaemOzay2aa0baaSqaaiabdQgaQjabdUgaRbqaaiabdsha0baaaaa@3182@, Sikq
 MathType@MTEF@5@5@+=feaafiart1ev1aaatCvAUfKttLearuWrP9MDH5MBPbIqV92AaeXatLxBI9gBaebbnrfifHhDYfgasaacPC6xNi=xH8viVGI8Gi=hEeeu0xXdbba9frFj0xb9qqpG0dXdb9aspeI8k8fiI+fsY=rqGqVepae9pg0db9vqaiVgFr0xfr=xfr=xc9adbaqaaeGacaGaaiaabeqaaeqabiWaaaGcbaGaem4uam1aa0baaSqaaiabdMgaPjabdUgaRbqaaiabdghaXbaaaaa@3154@ and Sjkt
 MathType@MTEF@5@5@+=feaafiart1ev1aaatCvAUfKttLearuWrP9MDH5MBPbIqV92AaeXatLxBI9gBaebbnrfifHhDYfgasaacPC6xNi=xH8viVGI8Gi=hEeeu0xXdbba9frFj0xb9qqpG0dXdb9aspeI8k8fiI+fsY=rqGqVepae9pg0db9vqaiVgFr0xfr=xfr=xc9adbaqaaeGacaGaaiaabeqaaeqabiWaaaGcbaGaem4uam1aa0baaSqaaiabdQgaQjabdUgaRbqaaiabdsha0baaaaa@315C@ are the frequencies and the position-specific score matrix (PSSM) scores of amino acid *k *and at position *i *of a template *q *and position *j *of a template *t*, respectively. For the secondary structure score (s_ij_), a positive score is added (subtracted) if the predicted secondary structure of the query protein at the position *i *is the same (different) type of secondary structure of the template protein at position *j*. Finally, the constant baseline score (*b*) is added to the alignment score.

The frequency matrices and PSSMs are generated by running PSI-BLAST [8] with default parameters except for the number of iterations (j = 11) and the E-value cutoff (h = 0.001). For each template of length n in the training set, alignments with the other templates in the training set are generated. Then, these alignments are transformed, respectively, into (n + 1)-dimensional feature vectors,

(*sa*^1^, *sa*^2^, ..., *sa*^*i*^, ..., *sa*^*n*^, *queary*_*lenth*)

where *sa*^*i *^is the profile-profile alignment score at position *i *of a given template [45] and *query_length *is the length of the query protein (Figure 1). If gaps occur, fixed negative scores are arbitrarily assigned. This is the modified version of [24]. The difference is that we use *query_length *instead of total alignment score. Since the size of the vector, *n *is dependent on the length of template protein, we make the same number of SVRs for all templates.

### SVR training

Only templates sharing at least the same fold with a target template are trained. To learn as many alignment examples as possible, 48 alignments are made per each pair of a query and a template (Table 2). Gap open penalty ranging from 5 to 13 is used; gap extension is one or two; baseline value is zero or one. The parameter for the predicted secondary structure information content is also varied. The input and the target of SVR are derived from the previous two sections. We would like to emphasize that there is no correct alignment example. Regression is basically a real value prediction. In training step for each input-target data of training sample, SVR models are trained with radial basis function (RBF) kernel without attempting serious performance optimization by SVMlight version 6.01 with the parameter gamma of 0.001 [46].

## Availability and requirements

The method is implemented in the platform-independent web server, FORECAST as a part. It is freely available without any restriction at 

## Authors' contributions

ML wrote the code for the analysis, carried out the training and testing SVRs, and drafted the manuscript. CSJ wrote the code for profile-profile alignment and implemented the code which generates input feature vectors for SVRs. DK participated in the design of the work and collaborated in writing the manuscript. All authors have read and approved the manuscript.

## Supplementary Material

Additional file 1 **Performance of SVR models at the family level**. (a) Correlations between observed and predicted MaxSub scores at the family level. Adjacent color bar shows the mapping of relative density. (b) Plot of frequency distribution. (c) Plot of MAE distribution. (d) Plot of NMAE distribution Click here for file

Additional file 2 **Performance of SVR models at the superfamily level**. (a) Correlations between observed and predicted MaxSub scores at the superfamily level. Adjacent color bar shows the mapping of relative density. (b) Plot of frequency distribution. (c) Plot of MAE distribution. (d) Plot of NMAE distribution Click here for file

Additional file 3 **Performance of SVR models at the fold level**. (a) Correlations between observed and predicted MaxSub scores at the fold level. Adjacent color bar shows the mapping of relative density. (b) Plot of frequency distribution. (c) Plot of MAE distribution. (d) Plot of NMAE distribution. Click here for file
